# Complete Genome Sequence of Helicobacter pylori Strain 3401, a Suitable Host for Bacteriophages KHP30 and KHP40

**DOI:** 10.1128/MRA.00647-21

**Published:** 2021-10-21

**Authors:** Michiko Takahashi, Yuki Matsumoto, Takako Ujihara, Hiromichi Maeda, Kazuhiro Hanazaki, Keizo Nagasaki, Hiroaki Takeuchi, Shigenobu Matsuzaki

**Affiliations:** a Faculty of Science and Technology, Kochi University, Nankoku, Kochi, Japan; b Science Research Center, Kochi University, Nankoku, Kochi, Japan; c Department of Surgery, Kochi Medical School, Kochi University, Nankoku, Kochi, Japan; d Faculty of Agriculture and Marine Science, Kochi University, Nankoku, Kochi, Japan; e Department of Medical Laboratory Sciences, Health and Science, International University of Health and Welfare Graduate School, Chiba, Japan; f Department of Medical Laboratory Science, Faculty of Health Sciences, Kochi Gakuen University, Kochi, Kochi, Japan; Loyola University Chicago

## Abstract

Helicobacter pylori 3401, isolated from a patient with duodenal ulcers in Japan, is susceptible to the bacteriophages KHP30 and KHP40. In this study, we report the complete genome sequence of H. pylori 3401. This study may lead to the establishment of phage therapy against H. pylori infection.

## ANNOUNCEMENT

Helicobacter pylori is a Gram-negative spiral bacterium that colonizes the human gastric mucosa and is estimated to infect half of the world’s population (1). With the emergence of antibiotic-resistant H. pylori strains, the development of bacteriophage therapy is desirable (2, 3). In 2012, two H. pylori bacteriophages (KHP30 and KHP40) that have a lytic effect on several strains of H. pylori were discovered (4, 5). H. pylori 3401 is used as a suitable host for these bacteriophages (4, 5). Here, we report the complete genome sequence of H. pylori 3401. This study has no ethical issues regarding research using humans or animals.

H. pylori 3401 was isolated from duodenal ulcer biopsy samples from a Japanese patient at Yamaguchi University Hospital, Yamaguchi, Japan, in 1991 (6). The isolated strain was cultured on BEV (Brucella supplemented with 10% [vol/vol] equine serum and 10 μg/ml vancomycin) agar plates under microaerobic conditions (10% CO_2_) at 37°C (5). A single colony was subcultured in BEV broth, and genomic DNA was extracted using the phenol-chloroform method.

DNA sequencing was conducted using an Illumina MiSeq platform (San Diego, CA, USA) with v2 chemistry (300-bp paired-end reads) and a Nanopore GridION X5 platform with a FLO-MIN106 R9.41 flow cell (Oxford Nanopore Technologies, Oxford, UK), after the sequencing libraries were prepared using a Nextera DNA Flex library prep kit (Illumina) and a ligation sequencing kit (SQK-LSK109, Oxford Nanopore Technologies), respectively. The Illumina reads were trimmed using fastp v0.20.1 (7) (parameters: ≥Q30 is qualified, ≥20 bp limited), and the Nanopore reads were trimmed using NanoFilt v2.7.1 (8) (parameters: ≥Q10 is qualified, ≥1,000 bp limited). The statistics of the raw/trimmed reads are shown in Table 1. Both sets of trimmed reads were hybrid-assembled using Unicycler v0.4.8 (9); then, the assembled contig was checked for other plasmids and contaminants using metaplasmid SPAdes v3.13.1 (10) and BlobTools v1.0 (11) with default parameters. The circular structure of this genome was also confirmed using Bandage v0.8.1 (12). The resulting circular contig was polished using Pilon v1.23 (13) with default parameters. The coverage of the genome was 47×. DFAST v1.2.4 (14) was used for auto-annotation.

**TABLE 1:** Statistics of raw reads and quality controlled reads

Type of reads	No. of sequences	Sum of length (bp)	Minimum length (bp)	Avg length (bp)	Maximum length (bp)	Nanopore *N*_50_ (bp)
Illumina_raw	795,261	122,023,362	35	153.4	156	
Illumina_trimmed	780,932	116,710,771	31	149.5	152	
Nanopore_raw	52,944	116,384,428	1	2,198.3	28,359	6,084
Nanopore_trimmed	21,908	59,325,730	1,000	2,707.9	27,925	6,673

The H. pylori 3401 assembly consisted of a circular chromosome of 1,591,853 bp (GC content, 39%). It contained 1,540 coding sequences (CDSs), four rRNAs, and 36 tRNA genes. Multilocus sequence typing (MLST) in PubMLST (https://pubmlst.org/) identified known allele numbers only for the *efp* gene as 2220, and all four sequence types containing allele 2220 in the *efp* gene (ST2789, ST2798, ST2824, and ST2845) were isolated in Japan, while previously undescribed alleles were detected for the genes *atpA*, *mutY*, *ppa*, *trpC*, *ureI*, and *yphC*. These results will contribute to elucidating the infection mechanism of bacteriophages KHP30 and KHP40 against H. pylori and help to develop therapeutic strategies.

### Data availability.

The complete genome sequence of H. pylori 3401 has been deposited in GenBank under the accession number AP024599. The raw data are available in the DDBJ Sequence Read Archive (DRA) under the accession number DRA011836.
